# Apigenin sensitizes BEL-7402/ADM cells to doxorubicin through inhibiting miR-101/Nrf2 pathway

**DOI:** 10.18632/oncotarget.18294

**Published:** 2017-05-30

**Authors:** Ai-Mei Gao, Xiao-Yu Zhang, Zun-Ping Ke

**Affiliations:** ^1^ Department of Pharmacy, The Fifth People's Hospital of Shanghai, Fudan University, Shanghai, China; ^2^ Department of Clinical Pharmacy, Shanghai General Hospital, Shanghai Jiaotong University School of Medicine, Shanghai, China; ^3^ Division of Gastrointestinal Surgery, Department of General Surgery, The Affiliated Huai'an Hospital of Xuzhou Medical University and The Second People's Hospital of Huai'an, Huai'an, China; ^4^ Department of Cardiology, The Fifth People's Hospital of Shanghai, Fudan University, Shanghai, China

**Keywords:** apigenin, microRNA-101, chemo-sensitization, Nrf2, hepatocellular carcinoma

## Abstract

Chemo-resistance is one of the main obstacle in hepatocellular carcinoma therapy. Apigenin as a natural bioflavonoid has been exhibited anti-cancer properties in various malignant cancers. The aim of this study is to evaluate the potential chemo-sensitization effect of apigenin in doxorubicin-resistant hepatocellular carcinoma cell line BEL-7402/ADM and to investigate its possible mechanism. We found that apigenin significantly reversed doxorubicin sensitivity and induced caspase-dependent apoptosis in BEL-7402/ADM cells. Furthermore, apigenin induced miR-101 expression, and overexpression of miR-101 mimicked the doxorubicin-sensitizing effect of apigenin. Importantly, we showed that miR-101 was able to target the 3′-UTR of Nrf2. The results suggested that apigenin sensitizes BEL-7402/ADM cells to doxorubicin through miR-101/Nrf2 pathway, which furtherly supports apigenin as a potential chemo-sensitizer for hepatocellular carcinoma.

## INTRODUCTION

Hepatocellular carcinoma (HCC), which is one of the most prevalent malignant diseases, remains the third leading cause of cancer-related deaths [1]. Although hepatic resection, chemotherapy, and liver transplantation are widely used to improve outcomes of patients with HCC, the mortality rate remains high. A major factor for this failure is chemo-resistance. Therefore, understanding the molecular mechanisms involved in the chemo-resistance of HCC may lead to improved clinical outcomes.

Apigenin (4′,5,7-trihydroxyflavone, APG), which is a natural bioflavonoid widely present in many fruits and vegetables, has been exhibited anti-cancer properties in various malignant cancers such as colon, skin, lung, prostate, and ovarian cancer [2]. APG exerts its anti-cancer effects by inhibiting tumor cell growth, invasion, and metastasis, and inducing cell cycle arrest and apoptosis [3]. Furthermore, combined therapy using APG with other chemotherapeutic drugs has been shown to enhance anticancer effects by blocking multiple signal transduction pathways such as nuclear factor-κb, PI3K-Akt and β-catenin [4, 5]. However, the molecular mechanisms of APG enhance sensitivity to chemotherapy drugs in HCC are still not fully understood.

MicroRNAs (miRNAs), which are a class of noncoding RNA with the length of 18–25 nucleotides, regulate protein expression post-transcriptionally by targeting the 3′-UTRs of target mRNA, resulting in inhibited translation or the degradation of the targeted mRNA [6]. Growing evidence indicates that miRNAs play key roles in the regulation of tumour cell proliferation, apoptosis, migration and invasion. Recently, association between miRNAs and chemo-sensitivity has been found to be involved in various human malignancies and attracted increasing attentions [7]. Among these, miR-101 has been found prevalently downregulated and plays a suppressive role in several types of cancer, including gastric cancer, colorectal cancer, lung cancer, melanoma, and so on [8, 9]. Furthermore, several studies have investigated the role of miR-101 in HCC. MicroRNA-101 suppresses migration and invasion via targeting vascular endothelial growth factor-C in hepatocellular carcinoma cells [10]. A previous study showed that miR-101 inhibited autophagy and enhanced cisplatin-induced apoptosis in HCC cells [11]. However, our understanding of the relationship between miR-101 and the sensitivity of HCC to doxorubicin (ADM)-based treatment remains largely limited.

Interestingly, in the present study, we found that miR-101 enhances the sensitivity of the human ADM-resistant hepatocellular carcinoma cell line BEL-7402/ADM to ADM, and miR-101 exerts its chemo-sensitization role by targeting Nrf2. In addition, we identified that APG sensitizes BEL-7402/ADM cells to ADM through inhibiting miR-101/Nrf2 pathway. Our results could furtherly support evaluation of APG as a potential chemo-sensitizer for HCC.

## RESULTS

### APG sensitizes BEL-7402/ADM cells to ADM

**Figure 1:**
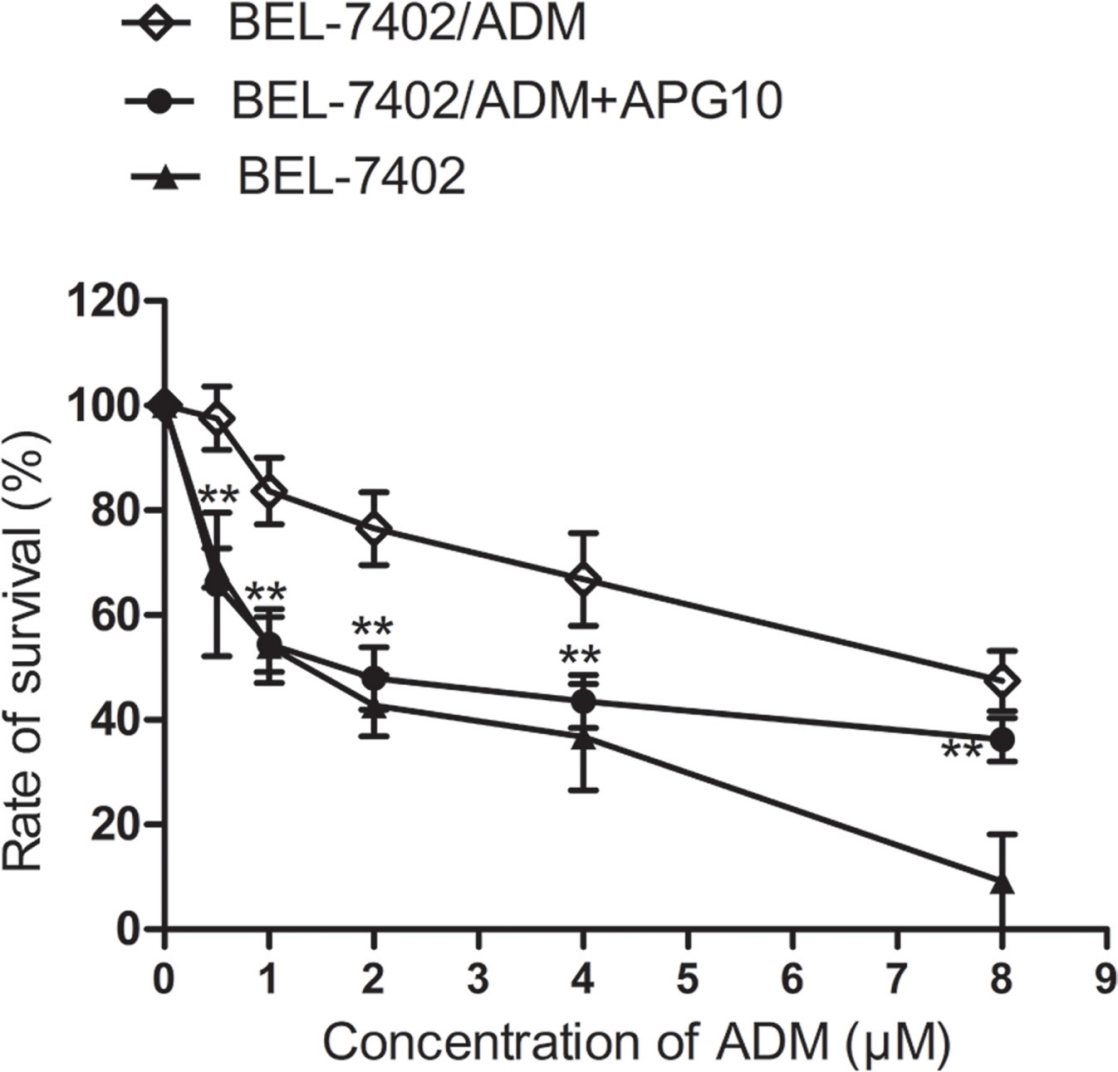
Effects of APG on ADM-induced cytotoxicity in BEL-7402/ADM cells BEL-7402/ADM cells were treated with indicated doses of ADM (0.5–8 μM) or ADM in combination with 10 μM APG for 48 h. The cell viability was detected by MTT assay. Data are expressed as mean ± SD (*n =* 3). Data are expressed as mean ± SD (*n =* 3). ^**^P < 0.01 vs the BEL-7402/ADM group.

To explore the potential role of APG in modulating ADM sensitivity of BEL-7402/ADM cells, we performed a survival analysis in BEL-7402/ADM cells. Cells were cultured in the presence of various doses of ADM or ADM in combination with 10 μM APG for 48 h. The cell survival was detected by MTT assay. As shown in Figure 1, BEL-7402/ADM cells exhibited a significant ADM resistance compared with its parental BEL-7402 cells, and treatment with APG could partially reversed this effect.

### APG enhances caspase 3-dependent apoptosis

**Figure 2:**
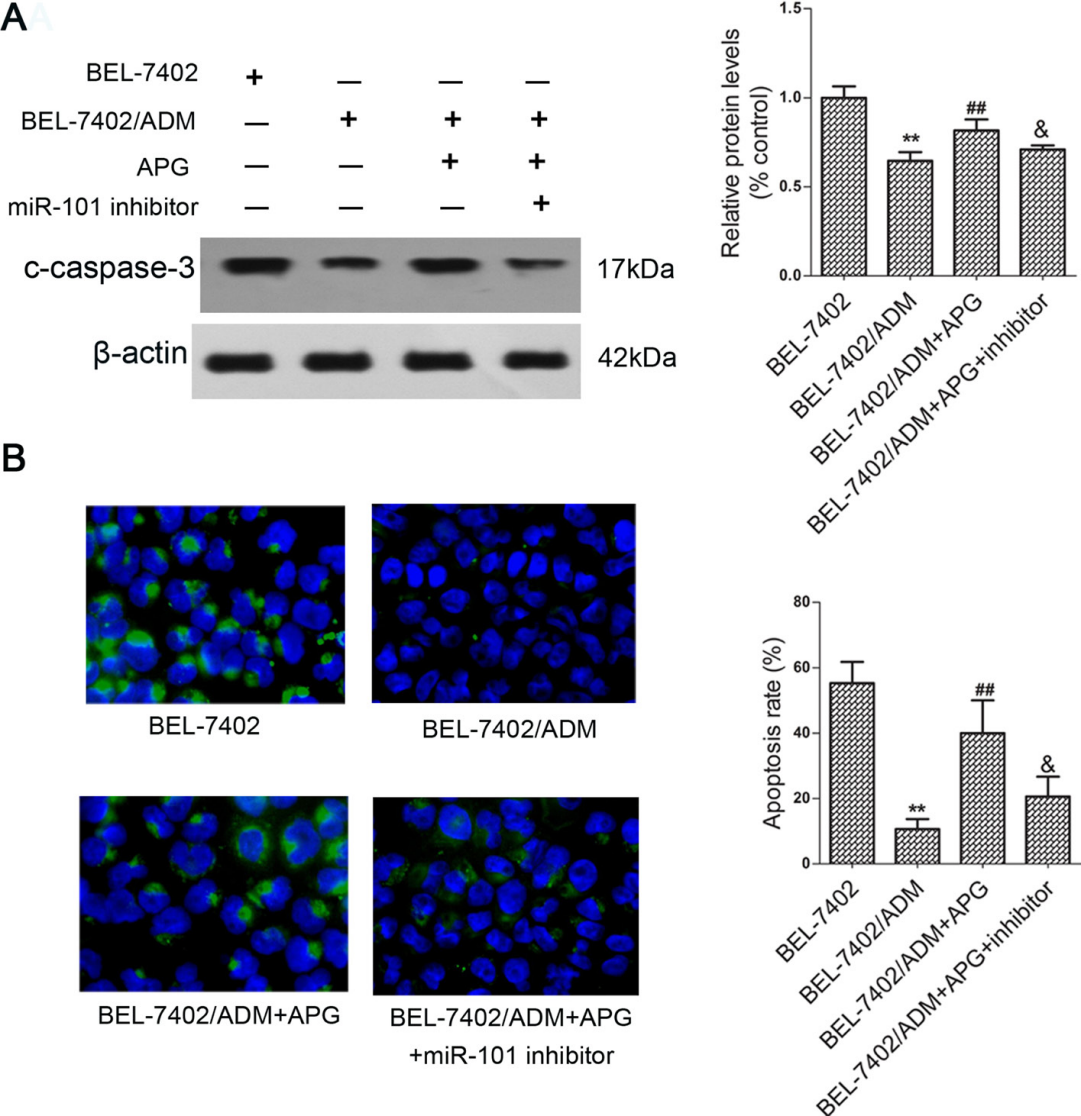
APG induces caspase-3 dependent apoptosis in BEL-7402/ADM cells Cells were treated with 8 μg/ml ADM or ADM in combination with APG (10 μM) for 48 h, and cleaved caspase-3 expression was detected by western blot (**A**). (**B**)The apoptosis of BEL-7402/ADM cells was determined by TUNEL assay. Data are expressed as mean ± SD (*n =* 3). ^**^P < 0.01 vs the BEL-7402 group; ^##^*P <* 0.01 vs the BEL-7402/ADM group; ^&^*P <* 0.05 vs the BEL-7402/ADM+APG group.

To further probe the regulatory mechanisms of APG in chemo-sensitization role, cleaved caspase-3 by western blot revealed that the protein level of c-caspase-3 in BEL-7402/ADM cells was obviously reduced comparing with the parental BEL-7402 cells. However, pretreatment with APG could partially restore this expression (Figure 2A). TUNEL assay confirmed that BEL-7402/ADM cells were resistant to ADM-induced apoptosis, which was then reversed by pretreatment with APG (Figure 2B). Interestingly, the effects of APG were attenuated by cotransfection with miR-101 inhibitor.

### MiR-101 mediates chemo-sensitization effect induced by APG

**Figure 3:**
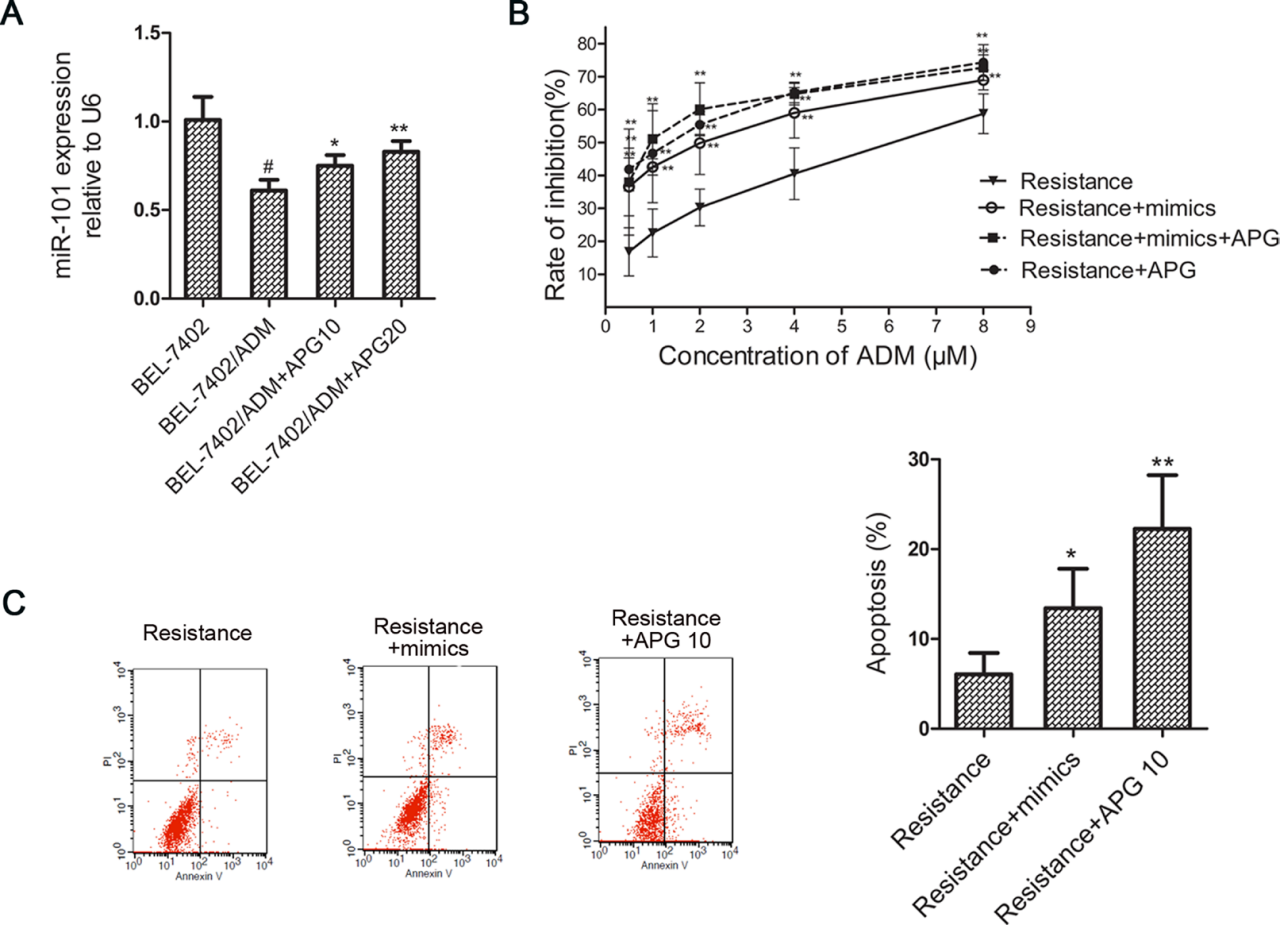
MiR-101 mediates APG induced ADM-sensitizing effect in BEL-7402/ADM cells (**A**) The miR-101 levels were detected in BEL-7402 and BEL-7402/ADM cells treated with APG by real-time PCR. (**B**) The effect of miR-101 mimics on cell viability in BEL-7402/ADM cells. Cells were transfected with miR-101 mimics and treated with different doses of ADM for 48 h. The cell viability was detected by MTT assay. (**C**) The effect of miR-101 mimics on cell apoptosis in BEL-7402/ADM cells was detected by FCM. Data are expressed as mean ± SD (*n* = 3). *P < 0.05, **P < 0.01 vs the BEL-7402/ADM group; ^#^*P* < 0.05 vs the BEL-7402 group.

Accumulating evidence indicates that miR-101 is closely associated with both cancer development and chemotherapy resistance [10, 11]. Next, we tested whether APG enhances ADM-induced cytotoxicity through inhibiting miR-101 expression. As shown in Figure 3A, the miR-101 levels in BEL-7402/ADM cells were downregulated compared with parental BEL-7402 cells and APG treatment restored its expression. This suggested that miR-101 might be involved in the control of ADM sensitivity by APG.

To confirm whether the chemo-sensitization by APG was miR-101 dependent, we tested the sensitivity of BEL-7402/ADM cells to ADM transfected with miR-101 mimics. As is shown in Figure 3B, BEL-7402/ADM cells that were transfected with the miR-101 mimics were much more sensitive to ADM than BEL-7402/ADM cells. The effect was similar to what we had observed upon APG treatment. However, the effect of APG was distinctly diminished in miR-101 mimics-transfected BEL-7402/ADM cells. These results indicated that miR-101 may mediate the ADM-sensitization effect induced by APG.

As shown in Figure 3C, transfection with miR-101 mimics significantly increased the percentage of apoptosis induced by ADM, and APG also markedly increased the cell apoptosis compared with the BEL-7402/ADM group. The result suggested that ADM-sensitization effect induced by APG may be concerned with miR-101 and apoptosis.

### Nrf2 is a direct target of miR-101

**Figure 4:**
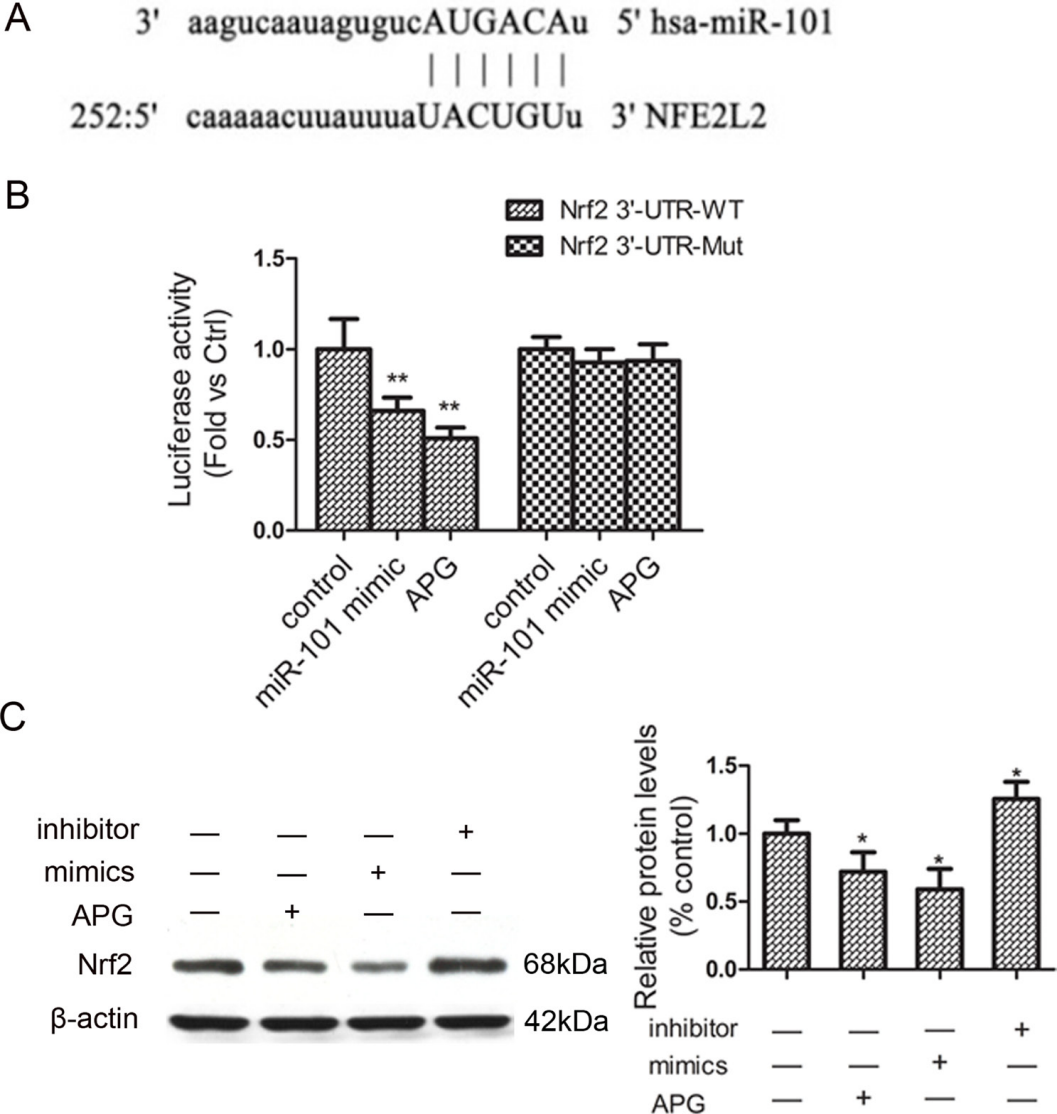
Nrf2 is a direct target of miR-101 in BEL-7402/ADM cells (**A**) The predicted binding site in the 3′-UTR of Nrf2 for miR-101. (**B**) The effect of miR-101 mimics and APG on the luciferase activity of the Nrf2 3′-UTR reporter. (**C**) The effect of miR-101 on the protein expression of Nrf2. Data are expressed as mean ± SD (*n =* 3). ^*^P < 0.05, ^**^P < 0.01 vs the BEL-7402/ADM group.

To further clarify the molecular mechanisms of miR-101 in ADM sensitivity, we used a miRNA target prediction program “miRanda” to predict the putative targets. Specifically, the 3′-UTR of Nrf2 mRNA contained a complementary site for miR-101 (Figure 4A). We deduced that Nrf2 might be involved in the ADM-sensitizing effect of miR-101 and APG. To confirm this, a luciferase reporter assay was performed. As shown in Figure 4B, miR-101 mimics reduced the activity of the luciferase reporter fused with the Nrf2 3′-UTR-WT by 36%, and APG treatment also markedly decreased the luciferase activity. The luciferase of reporter vector with the mutated Nrf2 3′-UTR was not affected by miR-101 mimics and APG. Furthermore, Western blot analysis revealed that the protein level of Nrf2 decreased after transfection with miR-101 mimics, whereas was increased by the miR-101 inhibitor compared with those in the BEL-7402/ADM group (Figure 4C). These data indicated that Nrf2 is directly and negatively regulated by miR-101. miR-101/Nrf2 axis may play a major role in chemo-sensitive effect of APG.

## DISCUSSION

APG, a potent dietary phytochemical, has been exhibited antioxidant, anti-inflammatory, and antitumor properties in various malignant cancers [2]. APG also exerts its potent chemo-sensitization activities due to its low intrinsic toxicity and differential effects in normal versus cancer cells [12]. In the present study, we reported that APG could sensitize BEL-7402/ADM cells to ADM by inducing caspase 3-dependent apoptosis. To investigate the mechanism of this effect, we found that upregulation of miR-101 confers the ADM-sensitizing role of APG, and Nrf2 functions as a targe of miR-101. Our results support further evaluation of APG as a potential chemo-sensitizer for HCC.

In this study, we observed that miR-101 was significantly downregulated in BEL-7402/ADM cells compared with the BEL-7402 cells. However, pretreatment with APG could restore its expression back to a relatively normal state. we also found that APG enhances the ADM sensitivity by inducing cell apoptosis in BEL-7402/ADM cells, whereas miR-101 mimics was able to exert effects similar to APG treatment, suggesting that miR-101 may confer the chemo-sensitization effect of APG. miR-101 has been suggested as a tumor suppressor, which regulates growth, apoptosis, migration and invasion of various tumor cells. For example, Wang L et al. reported that microRNA-101 suppresses progression of lung cancer through the PTEN/AKT signaling pathway by targeting DNA methyltransferase 3A [13]. Recently, Wu X et al. showed that miR-101-3p suppresses HOX transcript antisense RNA (HOTAIR)-induced proliferation and invasion through directly targeting SRF in gastric carcinoma cells [14]. Interestingly, miR-101 has also been implicated in osteosarcoma. miR-101 blocked the autophagy of osteosarcoma cells and thus enhanced osteosarcoma cell chemosensitivity [15]. In the present study, we reported for the first time that miR-101 was also downregulated in BEL-7402/ADM cells, which emphasized the importance of this microRNA in the pathogenesis of HCC.

A number of studies have suggested that the expression of some microRNAs is altered by APG, and these microRNAs yield powerful actions to confer the biological function of APG. For example, APG may improves glucose tolerance through suppression of matured miR103 expression levels [16]. APG inhibits hepatitis C virus replication by decreasing mature microRNA122 levels [17]. Noticeably, researches recently reported that APG and APG-rich diets exert effective anti-inflammatory activity *in vivo* by reducing LPS-induced expression of miR-155, thereby restoring immune balance [18]. Our work demonstrated for the first time that miR-101 was upregulated by APG in BEL-7402/ADM cells. This finding further expanded the multiple pharmacological mechanisms of APG. In this study, we identified that Nrf2, a transcription factor which serves as a cellular sensor for oxidative and electrophilic insults, is a functional target of miR-101. More importantly, our study identified a novel pathway that is involved in the pro-apoptotic process of APG for the first time, which extended our understanding on the drug-sensitizing effect of APG. However, as microRNA may not solely target a single gene, other possible targets may also participate in this process, which is worth our further research.

In conclusion, our research provides the first evidence that APG enhances the ADM sensitivity by altering the miR-101/Nrf2-related apoptosis pathway in HCC. Therefore, APG may be potentially used in combination with ADM to enhance the chemotherapeutic response in ADM-resistant patients.

## MATERIALS AND METHODS

### Cell lines and culture

The human HCC cells line BEL-7402 and ADM-resistant HCC cell line BEL-7402/ADM was purchased from Keygen Biotech Co., Ltd., (Nanjing, China). BEL-7402 cells were cultured in RPMI-1640 medium containing 10% fetal bovine serum. BEL-7402/ADM cells were cultured in the above-mentioned medium with addition of 2 μM ADM for at least 4 weeks prior to the experiment. Cells were maintained at 37°C in a humidified atmosphere containing 5% CO2.

### Cell viability assay

Cells were seeded into 96 well plates with the concentration of 4000 cells/well. At about 70% confluence, cells were treated with APG (10 μM), miR-101 mimics (50 nM), APG and miR-101 mimics in combination followed by 48h incubation in fresh culture medium containing ADM (0.5–8 μM). Then, wells were added 20 µl of 3-(4,5-dimethylthiazole-2-yl)-2,5-diphenyl tetrazolium bromide (MTT) solution (2mg/ml) and incubated for 4 h, and 200 µL dimethyl sulfoxide was added to each well to dissolve formed formazan crystals. The optical density was measured at 570 nm by a microplate reader.

### TUNEL assay

Apoptosis assay was determined by the transferase-mediated deoxyuridine triphosphate-biotin nick end labeling (TUNEL) assay and using the In Situ Cell Death Detection Kit (Roche, Mannheim, Germany) according to the manufacturer’s protocol. Then, cells were counterstained with 4’, 6-diamidino-2-phenylindole (DAPI, Roche). The TUNEL positive cells were scored in five independent samples for each group.

### Quantitative real-time polymerase chain reaction (qRT-PCR)

Total RNAs were isolated using TRIzol reagent (Invitrogen) and purified using an RNeasy Mini Kit (Qiagen, Germany) according to the manufacturer’s instructions. cDNA was synthesized from the total RNA by using PrimeScript RT reagent kit (TaKaRa, Japan). The PCR reactions were processed with the following specific primers: for miR-101, 5′-CGGCGGTACAGTA CTGTGATAA-3′ (forward) and 5′-CTGGTGTCGTGGAGTCGGCAATTC-3′ (reverse); and for U6, 5′-CTCGCTTCGGCAGCACA-3′ (forward) and 5′-AACGCTTCACGAATTTGCGT-3′ (reverse). The expressions of miR-101 were based on the 2-ΔΔCt method, using U6 as internal controls.

### Cell transfection

Upon reaching 60–70% confluence, the cells were transfected with 100 nM of miR-101 mimics/inhibitor (GenePharma, China) by using Lipofectamine 2000 reagent (Invitrogen, USA) according to the manufacturer’s instructions. The oligonucleotides were synthesized with the following sequences: miR-101 mimic, 5′-UACAGUACUGUGAUAACUGAA-3′; miR-101 inhibitor: 5′-UUCAGUUAUCACAGUACUGUA-3′.

### Annexin V-FITC/PI apoptosis assay

Apoptosis was assessed using an annexin V-fluorescein isothiocyanate (FITC)/PI apoptosis detection kit (Beyotime Institute of Biotechnology, China) according to the manufacturer’s instructions. Briefly, cells were collected, and resuspended in Annexin-binding buffer at a concentration of 1×10^6^ cells/mL. Then, cells were stained with Annexin V-FITC and PI at room temperature in the dark. After 15 min, the cells were analysed by flow cytometry.

### Dual-luciferase reporter assay

Wild type (WT) or Mutant (Mut) 3′-UTR of Nrf2 were cloned into the pGL3-Basic vector (Promega, USA) respectively, and then constructed pGL3/Nrf2-WT and pGL3/Nrf2-Mut recombinant vector after sequencing. BEL-7402/ADM cells cultured in 24-well plate were co-transfected with 100 nM of miR-101 mimic and pGL3/Nrf2-WT or pGL3/Nrf2-Mut using Lipofectamine 2000 reagent. The luciferase activity was measured after 48 h transfection using the dual-luciferase reporter assay system (Promega, USA).

### Western blot

Proteins were extracted with RIPA buffer, separated by sodium dodecyl-sulfate polyacrylamide gel electrophoresis and electro-transferred onto the polyvinylidene difluoride membranes. After successful transfer of proteins, the membrane was blocked with 5% non-fat milk for 2 h at 37°C and incubated with anti-Nrf2 and anti-c-caspase 3 monoclonal antibodies (Abcam, UK) overnight at 4°C. The membrane was then incubated with secondary antibody conjugated to horseradish peroxidase for 1h at 37°C. Bands were visualized with an enhanced chemiluminescence detection kit following the manufacturer’s instructions.

### Statistical analysis

Experiments were performed in triplicate and results were expressed as mean±SD. Statistical significance was determined by one-way analysis of variance. *P <* 0.05 was considered statistically significant.
